# Supplementary material for: Iterative improvement in the automatic modular design of robot swarms
Source: PeerJ Comput Sci. 2020 Dec 7;6:e322. doi: 10.7717/peerj-cs.322 (PMC7924708; doi:10.7717/peerj-cs.322)
Supplement: Supplemental Information 3 [file peerj-cs-06-322-s003.zip › argos3/doc/api/standalone/a00653_source.html]

ARGoS: plugins/simulator/visualizations/qt-opengl/qtopengl\_lua\_syntax\_highlighter.h Source File


- Main Page
- Related Pages
- Namespaces
- Classes
- Files

- File List
- File Members

# plugins/simulator/visualizations/qt-opengl/qtopengl\_lua\_syntax\_highlighter.h

Go to the documentation of this file.

```
00001 
00007 #ifndef QTOPENGL_LUA_SYNTAX_HIGHLIGHTER_H
00008 #define QTOPENGL_LUA_SYNTAX_HIGHLIGHTER_H
00009 
00010 namespace argos {
00011    class CQTOpenGLLuaSyntaxHighlighter;
00012 }
00013 
00014 #include <QHash>
00015 #include <QSyntaxHighlighter>
00016 #include <QTextCharFormat>
00017 
00018 class QTextDocument;
00019 
00020 namespace argos {
00021 
00022    class CQTOpenGLLuaSyntaxHighlighter : public QSyntaxHighlighter {
00023 
00024       Q_OBJECT
00025 
00026    public:
00027 
00028       CQTOpenGLLuaSyntaxHighlighter(QTextDocument* pc_text);
00029       virtual ~CQTOpenGLLuaSyntaxHighlighter() {}
00030 
00031    protected:
00032 
00033       void highlightBlock(const QString& str_text);
00034 
00035    private:
00036       
00037       struct SHighlightingRule
00038       {
00039          QRegExp Pattern;
00040          QTextCharFormat Format;
00041       };
00042       QVector<SHighlightingRule> m_vecHighlightingRules;
00043 
00044       QRegExp m_cCommentStartExpression;
00045       QRegExp m_cCommentEndExpression;
00046 
00047       QTextCharFormat m_cKeywordFormat;
00048       QTextCharFormat m_cSingleLineCommentFormat;
00049       QTextCharFormat m_cMultiLineCommentFormat;
00050       QTextCharFormat m_cQuotationFormat;
00051       QTextCharFormat m_cFunctionFormat;
00052    };
00053 
00054 }
00055 
00056 #endif
```

---

Generated on 10 Jul 2018 for ARGoS by 
 1.6.1
